# Suppression of gut dysbiosis by *Bifidobacterium longum* alleviates cognitive decline in 5XFAD transgenic and aged mice

**DOI:** 10.1038/s41598-019-48342-7

**Published:** 2019-08-14

**Authors:** Hae-Ji Lee, Kyung-Eon Lee, Jeon-Kyung Kim, Dong-Hyun Kim

**Affiliations:** Neurobiota Research Center, Department of Life and Nanopharmaceutical Sciences, College of Pharmacy, Kyung Hee University, 26, Kyungheedae-ro, Dongdaemun-gu, Seoul 02447 Korea

**Keywords:** Dysbiosis, Alzheimer's disease

## Abstract

To understand the role of commensal gut bacteria on the progression of cognitive decline in Alzheimer’s disease via the microbiota-gut-brain axis, we isolated anti-inflammatory *Bifidobacterium longum* (NK46) from human gut microbiota, which potently inhibited gut microbiota endotoxin production and suppressed NF-κB activation in lipopolysaccharide (LPS)-stimulated BV-2 cells, and examined whether NK46 could simultaneously alleviate gut dysbiosis and cognitive decline in male 5xFAD-transgenic (5XFAD-Tg, 6 months-old) and aged (18 months-old) mice. Oral administration of NK46 (1 × 10^9^ CFU/mouse/day for 1 and 2 months in aged and Tg mice, respectively) shifted gut microbiota composition, particularly Proteobacteria, reduced fecal and blood LPS levels, suppressed NF-κB activation and TNF-α expression, and increased tight junction protein expression in the colon of 5XFAD-Tg and aged mice. NK46 treatment also alleviated cognitive decline in 5XFAD-Tg and aged mice. Furthermore, NK46 treatment suppressed amyloid-β, β/γ-secretases, and caspase-3 expression and amyloid-β accumulation in the hippocampus of 5XFAD-Tg mice. NK46 treatment also reduced Iba1^+^, LPS^+^/CD11b^+^, and caspase-3^+^/NeuN^+^ cell populations and suppressed NF-κB activation in the hippocampus of 5XFAD-Tg and aged mice, while BDNF expression was increased. These findings suggest that the suppression of gut dysbiosis and LPS production by NK46 can mitigate cognitive decline through the regulation of microbiota LPS-mediated NF-κB activation.

## Introduction

Alzheimer’s disease (AD), which is the leading cause of dementia, is a highly prevalent, progressive, and neurodegenerative disorder in the elderly^1,2^. The main risk for AD is increasing aging^2^. The majority of AD patients are occurred in 65 and older and AD occurrence is estimated to be 6–8% in the aged population (>65 years of age)^3^. The representative markers of AD patients are the accumulation of insoluble amyloid-β (Aβ) and hyperphosphorylated tau in the brain^4^. Aβ is formed from amyloid precursor protein (APP) by the catalysis of β- and γ-secretases^5^. Aβ plaques, the aggregated complex of full length Aβ, Aβ40 and Aβ42, is accelerated by the mutation of APP and presenilin-1 (Psen-1) and activates glial cells to further stimulate proinflammatory cytokines such as IL-1β and TNF-α in the brain of AD patients and animal models such as 5xFAD-transgenic (5xFAD-Tg) mice, resulting in the neuronal damage and loss^6,7^. Therefore, Aβ plaque is a key constituent of AD-associated pathologies, including oxidative toxicity, inflammation, aberrant synaptic plasticity, and memory impairment^8,9^. 5XFAD-tg mice aggressively forms Aβ plaques at 2 months, impairs cognitive function at 4 months, and cause neuronal loss at 9 months of age, as AD patients^10,11^. These characteristics make 5xFAD-tg mice useful for evaluating the efficacy of anti-AD drugs.

Gut microbiota consist of >1000 species from relatively few phyla including Firmicutes, Bacteroidetes, and Proteobacteria^12^. Although gut microbiota composition is inter-individually variable, a number of their functions for maintaining health status are associated with the core microbiota^13^. The gut microbiota of AD patients exhibits the decreased α-diversity and is compositionally different from those of healthy individuals; in particular, the number of Bifidobacteria was lower in the gut microbiota of AD patients than in those of healthy people^14,15^. The gut microbiota composition of 5XFAD-Tg mice is different from that of conventional control mice: the Frirmicutes population including *Clostridium leptum* is increased in 5XFAD-Tg mice while the Bacteroidetes population is decreased^16^. The gut microbiota composition of aged people and mice was also different from those of children and young mice, respectively^17,18^. Moreover, the gut microbiota-originated endotoxin level was higher in aged people, aged mice, and 5XFAD-Tg mice than those in their controls, respectively^17–19^. Gut microbiota composition is influenced by glucocorticoids, adrenaline, and noradrenaline secreted through the hypothalamic pituitary adrenal (HPA) axis^20^. Gut microbiota stimulate the immune and central nervous systems (CNS) through the microbiota-gut-brain (MGB) axis: gut bacteria affect host health status through nutrients, xenobiotic metabolites, microbial byproducts, immune cytokines, neuroendocrine hormones, and neurotransmitters^20,21^. Therefore, gut microbiota disturbance (dysbiosis) is associated with gastrointestinal (GI) diseases such as inflammatory bowel disease and systemic diseases such as obesity, autoimmune arthritis, and neurological and psychiatric disorders. Gut microbiota disturbance-mediated GI inflammation accelerates the occurrence of memory impairment. Moreover, excessive production of gut microbiota LPS by stressors, such as immobilization and intraperitoneal injection of LPS, caused dysbiosis, colitis, and memory impairment in mice^22^. Therefore, alleviating gut microbiota lipopolysaccharide (LPS)-mediated GI inflammation may be beneficial for the therapy of AD.

Probiotics, including Lactobacilli and Bifidobacteria, are frequently used to inhibit the proliferation of pathogens, to activate non-specific immune responses in their hosts, to improve anti-metabolic disorders, and to alleviate neuropsychiatric disorders^23–25^. *Lactobacillus plantarum* C29 alleviates the cognitive decline in aged rats and mitigated LPS- or scopolamine-induced memory impairment in mice^19,26^. *Bifidobacterium breve* A1 prevents Aβ-induced cognitive impairment by suppressing the hippocampal expressions of inflammation and immune-reactive genes in mice^27^. Furthermore, the mixture of Lactobacilli and Bifidobacteria (*Lactobacillus acidophilus, Lactobacillus fermentum, Bifidobacterium lactis*, and *Bifidobacterium longum*) improves Aβ-induced memory deficit in rats^28^. Other mixture of *Lactobacillus acidophilus, Lactobacillus casei, Bifidobacterium bifidum*, and *Lactobacillus fermentum* alleviates cognitive decline in the AD patients^29^. Nevertheless, whether Bifidobacteria known as commensal gut bacteria can attenuate the cognitive decline and what are their ameliorating mechanisms against memory impairment remain elusive.

Therefore, to understand the role of commensal gut Bifidobacteria in the occurrence of AD, we isolated anti-inflammatory *Bifidobacterium longum* NK46, which suppressed human gut microbiota LPS production and NF-κB activation in LPS-stimulated microglial BV-2 cells, from healthy human fecal microbiota and examined whether NK46 could alleviate cognitive decline and gut microbiota dysbiosis in 5XFAD-Tg and aged mice.

## Results

### *Bifidobacterium longum* suppressed gut microbiota LPS production and LPS-induced NF-κB activation in BV-2 cells

**Figure 1:**
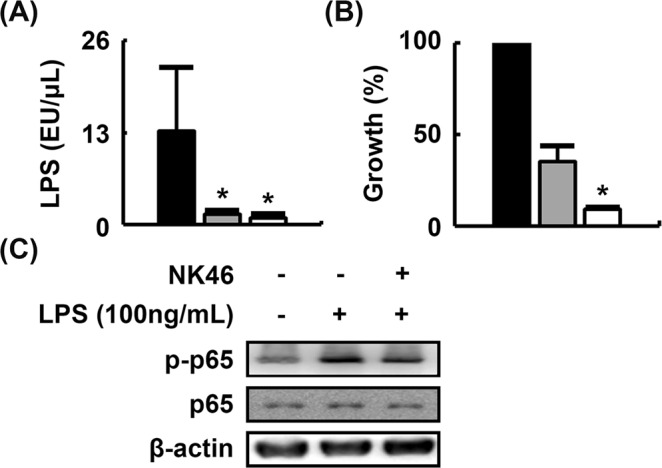
NK46 inhibited LPS production in mouse gut microbiota and NF-κB activation in LPS-stimulated microglial BV-2 cells. (**A**) Effect on LPS production. (**B**) Effect on fecal bacteria growth. Fecal bacterial cells (1 × 10^5^ colony forming unit [CFU]/mL) were anaerobically incubated in 10 mL of general anaerobic medium (GAM) broth with NK46 (black, vehicle alone; gray, 1 × 10^5^ CFU; white, 1 × 10^6^ CFU/mL) for 24 h. Number of fecal bacteria were counted in the GAM agar plate. (**C**) Effect on LPS-induced NF-κB activation in BV-2 cells. BV-2 cells (5 × 10^5^ cells/mL) were incubated with LPS in the absence or presence of NK46 (1 × 10^5^ CFU/mL). All data were expressed as mean ± SD (n = 4). ^*^p < 0.05 vs. control group treated with vehicle alone.

We examined the inhibitory effects of 25 Lactobacilli and 25 Bifidobacteria strains on gut microbiota LPS production. Of these, NK46 most potently inhibited LPS production and fecal bacterial growth (Fig. 1A,B). NK46 also potently inhibited LPS-induced NF-κB activation in microglial BV-2 cells (Fig. 1C). NK46 was identified as *Bifidobacterium longum*, based on the results of Gram staining, API 20A Kit (bioMerieux, Seoul, Korea), and 16S rDNA sequencing (ABI 3730XL DNA analyzer, Thermo Fisher Scientific Inc, Waltham, MA, USA).

### NK46 shifted gut microbiota composition and suppressed gut microbiota LPS production and colitis marker expression in 5XFAD-Tg mice

**Figure 2:**
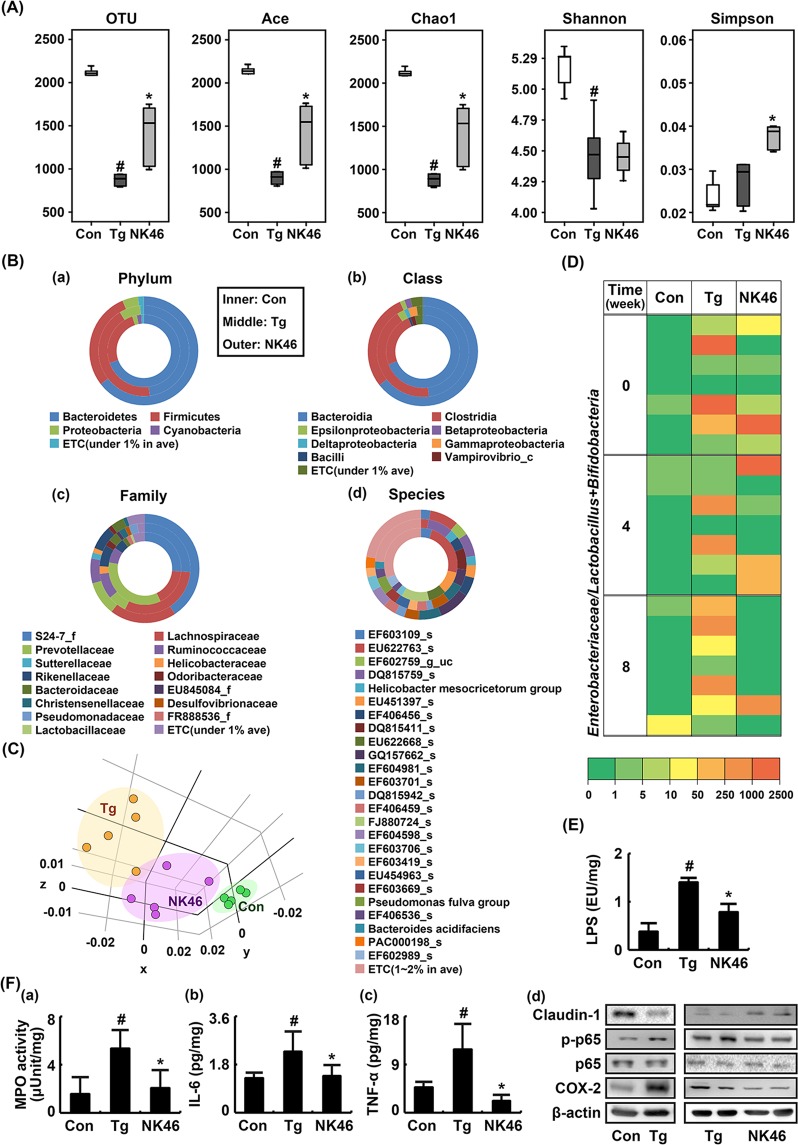
NK46 modified the gut microbiota composition in 5XFAD Tg mice. (**A**) Effect on the number of sequences analyzed, operational taxonomic units (OTUs), abundance-based coverage estimator (ACE), Chao1, Shannon, and Simpson. (**B**) Effect on the fecal microbiota composition: phylum (a), class (b), family (c), and species (d). (**C**) Principal coordinate analysis (PCoA) plot. The plot shows the clustering pattern among control (Con), vehicle- (Tg), and NK46-treated Tg mice (NK46) based on weighted pairwise Fast UniFrac analysis (n = 5). (**D**) Effect on the ratio of Enterobacteriaceae populations to Bifidobacteria plus Lactobacilli populations, assessed by the selective media (n = 6). (**E**) Effect on the fecal LPS levels. (**F**) Effect on the myeloperoxidase (MPO) activity (a) and IL-6 (b), TNF-α (c), claudin-1, and COX-2 expression, and NF-κB activation (d) in the colon. IL-6 and TNF-α levels were measured using ELISA and COX-2, claudin-1, p65, p-p65 were using immunoblotting. Test agent (Tg, vehicle alone; NK46, 1 × 10^9^ CFU/mouse/day) was orally administered for 2 months in Tg mice. Control mice (Con) were treated with vehicle alone. All data were expressed as mean ± SD (n = 6). ^#^*p* < 0.05 vs. Con group. *p < 0.05 vs. Tg group.

To examine whether NK46 could fine-tune gut microbiota composition, we orally gavaged NK46 in 5XFAD-Tg mice and measured the fecal microbiota composition using pyrosequencing (Fig. 2). Bacterial richness and α-diversity were significantly lower in 5XFAD-Tg mice than in control mice, as demonstrated by the number of sequences analyzed, estimated operational taxonomic unit (OTU) richness, abundance-based coverage estimator (ACE), Chao1, Shannon, and Simpson. (Fig. 2A). However, NK46 treatment increased bacterial richness and α-diversity. Comparing the results of taxonomy-based analysis between 5XFAD-Tg mice treated and not treated with NK46, it showed that the fecal microbiota composition of 5XFAD-Tg mice was significantly different from that of NK46-treated mice (Fig. 2B). At the phylum level, 5XFAD-Tg mice exhibited Proteobacteria and Firmicutes populations more abundantly than control mice, as previously reported^19^, while the Bacteroidetes population was lower in 5XFAD-Tg mice. Treatment with NK46 in 5XFAD-Tg mice decreased the populations of Firmicutes and Proteobacteria and increased the population of Bacteroidetes. At the class level, 5XFAD-Tg mice exhibited Clostridia, and δ-, γ-, and ε-Proteobacteria populations more abundantly than control mice, while Bacteroidia and Bacilli populations were lower. Treatment with NK46 in 5XFAD-Tg mice increased Bacteroidia population and suppressed Clostridia and δ-, γ-, and ε-Proteobacteria populations. At the family level, Lachnospiraceae, Ruminococaceae, Helicobacteriaceae, and Pseudomonadaceae populations were higher in 5XFAD-Tg mice than in control mice. Prevotellaceae populations were lower in 5XFAD-Tg mice than in control mice. However, treatment with NK46 reduced Ruminococaceae, Lachnospiraceae, Helicobacteriaceae, and Pseudomonadaceae populations and increased the Prevotellaceae population. At the species level, EF603109_s, EU622763_s, and EU622668_s populations were lower in 5XFAD-Tg mice than in control mice while DQ815759_s, *Helicobacter mesocricetorum* group, and DQ815411_s populations were higher. However, treatment of 5XFAD mice with NK46 increased EF603109_s and EU622763_s populations and reduced DQ815411_s population. Furthermore, NK46 treatment increased β-diversities (Fig. 2C). To understand the balance of beneficial to harmful bacteria in the gut microbiota composition, we investigated the ratio of live Enterobacteriaceae to Bifidobaceria plus Lactobacilli (E/BL) by using selective media, glucose blood liver (BL) and deoxycholate hydrogen sulfide lactose (DHL) agar plates (Fig. 2D). The E/BL ratio was higher in 5XFAD-Tg mice than in control mice. However, NK46 treatment reduced the E/BL ratio. Furthermore, treatment with NK46 inhibited fecal bacterial LPS production in 5XFAD-Tg mice (Fig. 2E).

Next, we examined whether NK46 treatment could suppress gut inflammation in 5XFAD-Tg mice (Fig. 2F). Myeloperoxidase activity and NF-κB activation significantly increased in the colons of 5XFAD-Tg mice compared to those of the control mice. However, oral administration of NK46 suppressed myeloperoxidase activity and IL-6 and TNF-α expression in the colon of 5XFAD-Tg mice (Fig. 2F(a–c)). NK46 treatment also inhibited NF-κB activation and COX-2 expression and increased the expression of claudin-1, a tight junction protein, in the colon of 5XFAD-Tg mice (Fig. 2F(d)).

### NK46 attenuated cognitive decline in 5XFAD-Tg mice

**Figure 3:**
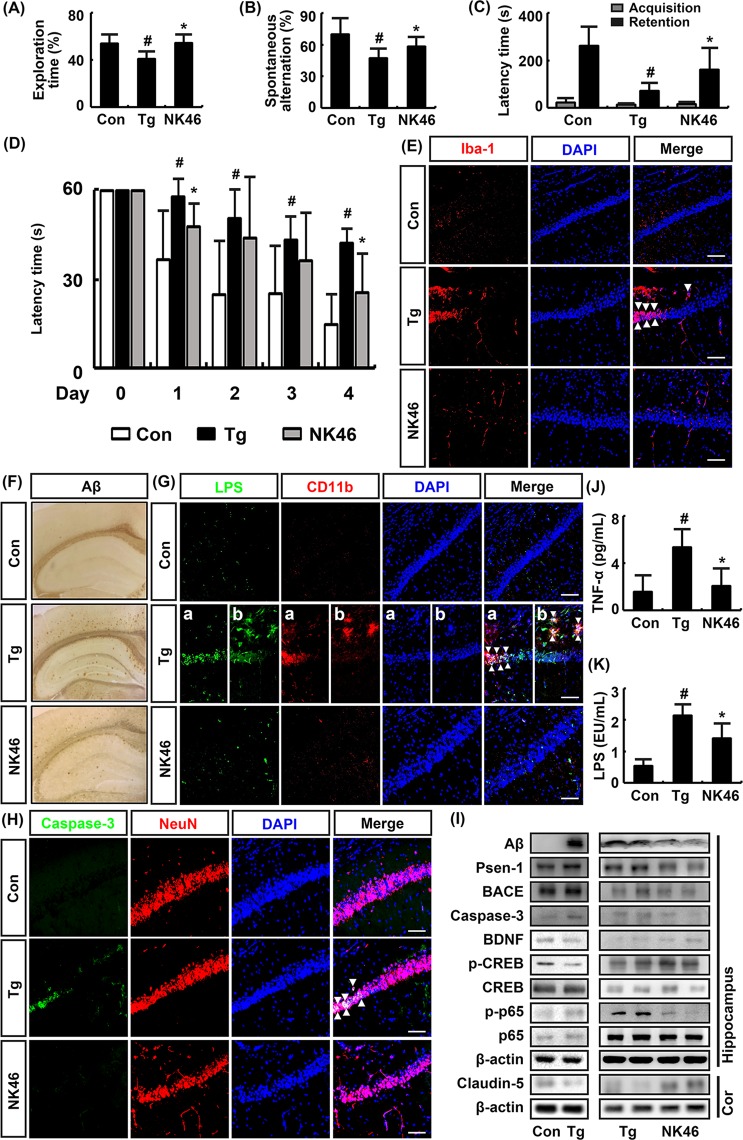
NK46 attenuated cognitive decline in 5XFAD-Tg mice. Effects on exploration time in novel object recognition task (**A**), spontaneous alteration in Y-maze task (**B**), latency time in passive avoidance task (**C**), and latency time in the Morris water maze tasks (**D**). (**E**) Effects on Iba1^+^ (activated microglia) population in the hippocampus, assessed by a confocal microscope. (**F**) Effect on the Aβ expression in the brain. (**G**) Effect on LPS^+^/CD11b^+^ in the pyramidal (a) and stratum oriens regions (b) of hippocampus. (**H**) Effect on caspase-3^+^/Neu^+^ (apoptotic neuron cell) populations. Bar indicates 0.1 mm. (**I**) Effect on BDNF, BACE1, Psen1, caspase-3, and claudin-5 expression, NF-κB and CREB activation, assessed by immunoblotting. Effect on TNF-α (**J**) and LPS levels (**K**) in the blood, assessed by ELISA. Test agent (Tg, vehicle alone; NK46, 1 × 10^9^ CFU/mouse/day) was orally administered for 2 months in Tg mice. Control mice (Con) were treated with vehicle alone. All data were expressed as mean ± SD (n = 6). ^#^*p* < 0.05 vs. Con group. ^*^p < 0.05 vs^.^ Tg group.

Next, we investigated whether NK46 could mitigate cognitive decline in 5XFAD-Tg mice in Y-maze, passive avoidance, novel object recognition, and Morris water maze tasks (Fig. 3). The cognitive function of 8 months-old 5XFAD-Tg mice was significantly impaired compared with that of 8 months-old control mice (Fig. 3A–D). Aβ plaques significantly accumulated in the brain cortex and hippocampus of Tg mice compared with those of control mice (Fig. 3F). Furthermore, caspase-3^+^/NeuN^+^ (apoptotic neuron cell), Iba+ (activated microglia), and LPS^+^/CD11b^+^ (LPS-phagocytic cell) populations were significantly increased in the pyramidal and Aβ-accumulated regions of the hippocampus (Fig. 3E,G). When NK46 was orally gavaged for 8 weeks in 6-month-old 5XFAD-Tg mice, as previously reported^19^, NK46 treatment significantly alleviated the progression of cognitive decline (Fig. 3A–D). NK46 treatment also significantly suppressed the accumulation of Aβ plaques and populations of Iba^+^, LPS^+^/CD11b^+^, and caspase-3^+^/NeuN^+^ cells in the hippocampus (Fig. 3E–H). BNDF and claudin-5 expression and CREB phosphorylation were significantly suppressed in the hippocampus of 5XFAD-Tg mice compared with those in control mice (Fig. 3I). However, treatment of 5XFAD-Tg mice with NK46 increased BDNF expression and CREB phosphorylation and inhibited NF-κB activation. NK46 treatment also suppressed Aβ, BACE1, Psen-1 (γ-secretase-1), and caspase-3 expression in the hippocampus of 5XFAD-Tg mice. Furthermore, NK46 treatment reduced TNF-α and LPS levels in the blood of 5XFAD-Tg mice (Fig. 3J,K).

### NK46 shifted gut microbiota composition and suppressed gut microbiota LPS production and colitis marker expression in aged mice

**Figure 4:**
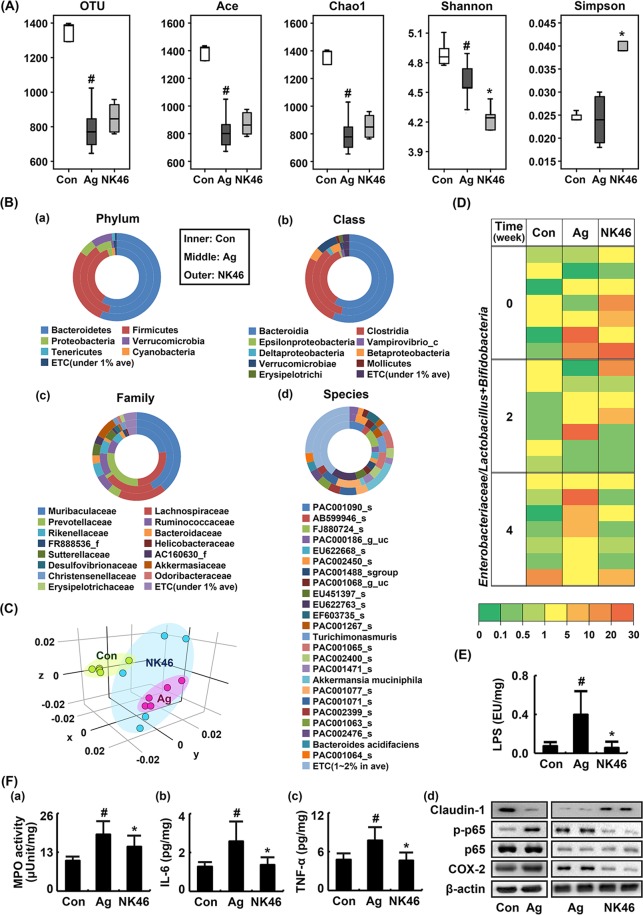
NK46 modified the gut microbiota composition in aged mice. (**A**) Effect on the number of sequences analyzed, operational taxonomic units (OTUs), abundance-based coverage estimator (ACE), Chao1, Shannon, and Simpson. (**B**) Effect on the fecal microbiota composition: phylum (a), class (b), family (c), and species (d). (**C**) Principal coordinate analysis (PCoA) plot. The plot shows the clustering pattern among control (Con), vehicle- (Ag), or NK46-treated aged mice (NK46) based on weighted pairwise Fast UniFrac analysis (n = 5). (**D**) Effect on the ratio of Enterobacteriaceae populations to bifidobacteria plus lactobacilli populations, assessed by the selective media BL and DHL agar plates (n = 6). (**E**) Effect on the fecal LPS levels. (**F**) Effect on the myeloperoxidase (MPO) activity (a) and IL-6 (b), TNF-α (c), claudin-1 and COX-2 expression, and NF-κB activation (d) in the colon. IL-6 and TNF-α levels were measured using ELISA and COX-2, claudin-1, p65, p-p65 were using immunoblotting. Test agent (Ag, vehicle alone; NK46, 1 × 10^9^ CFU/mouse/day) was orally administered for 1 month in aged mice. Control mice (Con) were treated with vehicle alone. All data were expressed as mean ± SD (n = 6). ^#^*p* < 0.05 vs. Con group. ^*^p < 0.05 vs. Ag group.

To evaluate whether short-term treatment with NK46 could also modify gut microbiota composition, we orally gavaged NK46 for 4 weeks in aged mice and measured the fecal microbiota composition of adult and aged mice treated with or without NK46 using pyrosequencing (Fig. 4). Bacterial richness and α-diversity were significantly lower in aged mice than in adult control mice (Fig. 4A). Treatment of aged mice with NK46 did not significantly increase α-diversity. At the phylum level, aged mice exhibited the Proteobacteria population more abundantly than control mice (Fig. 4B), as previously reported^18^. However, NK46 treatment reduced Proteobacteria population and increased Verrucomicrobia and Tenericutes populations. At the class level, β- and δ-Proteobacteria and Verrucomicrobiae populations were higher in aged mice than in control mice while ε-Proteobcteria and Vampirovibrio_c populations were lower. However, NK46 treatment suppressed δ-Proteobacteria population. At the family level, Muribacculaceae, Ruminococcaceae, Sutterellaceae, and Christensenellaceae populations were higher in aged mice than in control mice. Lachnospiraceae, Prevotellaceae, and Bacteroidaceae populations were lower in aged mice. However, treatment of aged mice with NK46 suppressed Muribacculaceae and Ruminococcaceae populations and increased Provotellaceae and Bacteroidaceae populations. At the species level, *Bacteroides* AB599946_s, *Paraprevotella* FJ880724_s, and *Prevotella* EU622763_s populations were lower in aged mice than in control mice while *Muribaculum* PAC001077_s, *Turicimonas muris*, and PAC000186_g PAC001065_s populations were higher. However, treatment of aged mice with NK46 increased PAC001077_s and *Turicimonas muris*, populations. NK46 treatment weakly, not significantly, increased α- and β-diversities (Fig. 4A,C). The ratio of E/BL was higher in aged mice than in control mice, assessed by culturing BL and DHL agar plates. However, NK46 treatment suppressed the E/BL ratio (Fig. 4D). Furthermore, treatment with NK46 inhibited fecal bacterial LPS production in 5XFAD-Tg mice (Fig. 4E).

Next, we examined whether NK46 could suppress gut inflammation in aged mice. Myeloperoxidase activity and NF-κB activation significantly increased in the colons of aged mice more potently than in those of control mice (Fig. 4F). However, oral administration of NK46 suppressed myeloperoxidase activity and TNF-α and IL-6 expression in the colon of aged mice (Fig. 4F(a–c)). NK46 treatment also inhibited NF-κB activation and COX-2 expression and increased claudin-1 expression in the colon of aged mice (Fig. 4F(d)).

### NK46 attenuated cognitive decline in aged mice

**Figure 5:**
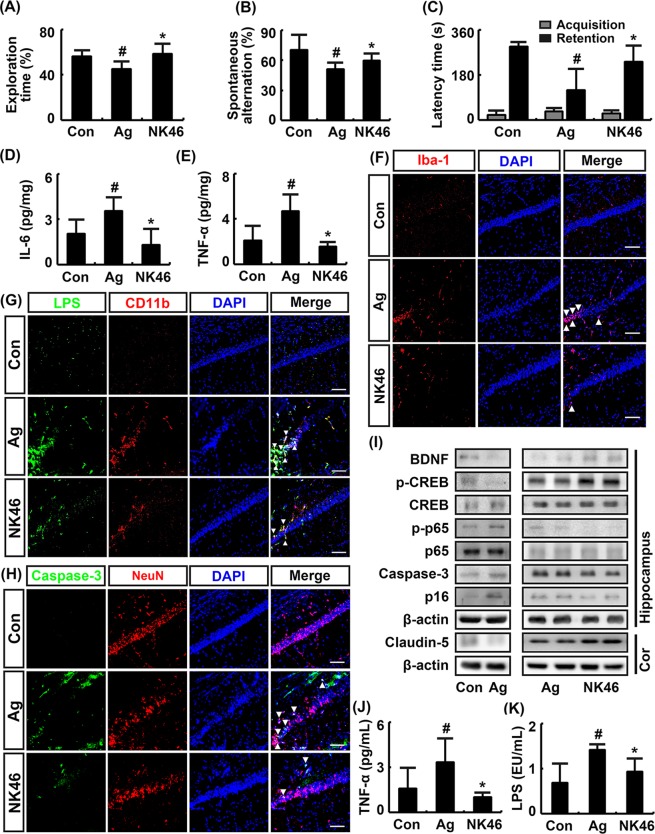
NK46 attenuated cognitive decline in aged mice. Effects on exploration time in novel object recognition task (**A**), spontaneous alteration in Y-maze task (**B**), and latency time in passive avoidance task (**C**). Effect on IL-6 (**D**) and TNF-α (**E**) expression in hippocampus, assessed by ELISA. Effect on Iba1^+^ (**F**), LPS^+^/CD11b^+^ (**G**), and caspase-3^+^/Neu^+^ cell populations (**H**) in hippocampus, assessed by a confocal microscope. Bar indicates 0.1 mm. (**I**) Effects on BDNF, caspase-3, and claudin-5 expression, NF-κB and CREB activation, assessed by immunoblotting. Effect on TNF-α (**J**) and LPS levels (**K**) in the blood. Test agent (Ag, vehicle alone; NK46, 1 × 10^9^ CFU/mouse/day) was orally administered for 1 month in aged mice. Control adult mice (Con) were treated with vehicle alone. All data were expressed as mean ± SD (n = 6). ^#^*p* < 0.05 vs. Con group. ^*^p < 0.05 vs. Ag group.

To understand whether NK46 could alleviate aging-dependent cognitive decline, we examined the effect of NK46 in aged mice in the novel object recognition, Y-maze, and passive avoidance tasks (Fig. 5). The cognitive function of 19-month-old (aged) mice was significantly impaired compared with that of 7-month-old (adult) mice (Fig. 5A–C). Oral administration of NK46 for 1 month in 18-month-old mice significantly alleviated aging-dependent cognitive decline. NK46 treatment also suppressed aging-induced TNF-α and IL-6 expression in the hippocampus (Fig. 5D,E). Aging also significantly increased Iba^+^, LPS^+^/CD11b^+^, and caspase-3^+^/NeuN^+^ cell populations in hippocampus (Fig. 5F–H). However, treatment of aged mice with NK46 significantly suppressed the infiltration of Iba^+^, LPS^+^/CD11b^+^, and caspase-3^+^/NeuN^+^ cells into the hippocampus. Aging also suppressed BNDF and claudin-5 expression and CREB phosphorylation and induced NF-κB activation and caspase-3 and p16 expression in the hippocampus (Fig. 5I). However, NK46 treatment alleviated aging-dependent suppression of BDNF and claudin-5 expression and CREB phosphorylation and induction of NF-κB activation and p16 expression. Treatment of aged mice with NK46 also suppressed blood TNF-α and LPS levels (Fig. 5J,K).

## Discussion

Aging is strongly associated with inflammation. Aging-dependent exposure to chronic, low-grade inflammation, termed “inflammaging”, triggers aggressive neurodegenerative diseases such as AD^30,31^. The pathological hallmarks of AD are Aβ plaques and hyper-phosphorylated tau tangles, which are accelerated by microbial infection^30,32^. Moreover, aged people and mice exhibit altered gut microbiota composition compared to children/adults and young mice, respectively^17,33^. The *Bifidobacteria* population is decreased in the elderly^34^. Both *Bifidobacteria* and *Lactobacilli* populations are lower in elderly individuals than in adults, whereas there are no differences in *Bacteroides* and *Eubacterium* levels^35^. The gut microbiota composition of the elderly is extremely variable between individuals^36^. Elderly subjects exhibit high populations of *Escherichia coli* and Bacteroidetes^37^. The Firmicutes population and fecal LPS are significantly higher in aged mice than adults and Proteobacteria population and fecal LPS production are significantly higher in 5XFAD-Tg mice^19^. 5XFAD-Tg and aged mice exhibit altered gut microbiota composition, as well as increased proinflammatory cytokine expression in the GI tract^18,19^. These results suggest that the excessive production of gut bacterial LPS by gut dysbiosis may cause GI inflammation. Jang *et al*. reported that GI inflammation by 2, 4, 6-trinitrobenzenesulfonic acid deteriorated cognitive function with gut dysbiosis^29^. These results suggest that GI inflammation can increase memory impairment.

In the present study, we also found that 5XFAD-Tg and aged mice exhibited the Proteobacteria population more abundantly than adult control mice. Furthermore, the production of gut bacterial LPS were higher in the colon fluid and the expression of tight junction protein such as claudin was lower in the colon of 5XFAD-Tg and aged mice than in control mice. NF-κB activation and COX-2 and TNF-α expression were higher in the colon of 5XFAD-Tg and aged mice than in the colon of adult mice. We also found that blood LPS levels were higher in 5XFAD-Tg and aged mice than in control mice. Jang *et al*. reported that GI inflammation increased the absorption of gut microbiota LPS into the blood in mice and LPS treatment caused endotoxemia as well as colitis in mice^22^. In the present study, Tg mice exhibited excessive Aβ plaques and increased gut Proteobacteria and Firmicutes populations than aged mice, while aged mice induced the p16 expression and ration of Enterobacteriaceae population to Lactobacilli plus Bifidobacteria population more potently than 5XFAD-Tg mice. These results suggest that the gut microbiota of Tg and aged mice excessively induce LPS production and the chronic exposure to excessive gut microbiota LPS can cause endotoxemia through the acceleration of GI inflammation.

Moreover, we found that NF-κB activation and caspase-3^+^/NeuN^+^ (apoptotic neuron cell), Iba^+^ (activated microglia), and LPS^+^/CD11b^+^ (LPS-phagocytic cell) populations were increased in the hippocampus of 5XFAD-Tg and aged mice. These results suggest that aging and hippocampal Aβ plaque accumulation can cause hippocampal inflammation via the activation of microglia cells by gut microbiota endotoxins such as LPS. However, the expression of BDNF, which maintains synaptic plasticity and memory storage in the hippocampus^38,39^, and claudin-5, which is one of tight junction protein in the brain^40^, was suppressed in these mice. Brain inflammation causes the suppression of BDNF and caludin-5 expression^38,40^. These results suggest that hippocampal inflammation in 5XFAD-Tg and aged mice can be accelerated by the suppression of tight junction protein expression in the brain as well as GI tract. In the present study, we found that BDNF expression and cognitive function were suppressed in 5XFAD-Tg and aged mice compared to those in control mice. Devi and Ohno reported that the formation of Aβ plaques reduced BDNF levels in 5XFAD-Tg mice^41^. Rangasamy *et al*. also reported that the overexpression of Aβ proteins increased NF-κB activation in 5XFAD-Tg mice^42^. Gut microbiota LPS levels in the intestine fluid and NF-κB activation in the hippocampus are higher in 5XFAD-Tg mice than in control mice^19^. LPS levels in the colon fluid and blood and NF-κB activation in the hippocampus are also higher in aged mice than in young mice^18^. These results suggest that hippocampal inflammation can suppress BDNF expression, resulting in memory impairment through the suppression of NF-κB-mediated BDNF expression.

However, treatment with NK46, which suppressed gut microbiota LPS production and LPS-induced NF-κB activation *in vitro*, suppressed NF-κB activation, COX-2 expression, and myeloperoxidase activity in the colon of 5XFAD-Tg and aged mice and increased tight junction protein expression. NK46 treatment reduced gut microbiota LPS production and Proteobacteria population in 5XFAD-Tg and aged mice. These results suggest that NK46 can alleviate Aβ- and aging-induced GI inflammation through the regulation of gut microbiota composition and LPS production.

NK46 treatment also suppressed LPS levels in the blood and feces of 5XFAD-Tg and aged mice and alleviated cognitive decline in 5XFAD-Tg and aged mice. NK46 treatment suppressed NF-κB activation, Aβ, caspase-3, β-secretase, and γ-secretase expression, Aβ plaque accumulation, and the number of caspase-3^+^/NeuN^+^ (apoptotic neuron cells), Iba^+^ (activated microglia), and LPS^+^/CD11b^+^ (LPS-phagocytic) cells and increased BDNF expression in the hippocampus of 5XFAD-Tg and aged mice. β- and γ-Secretases and caspase-3 catalyze Aβ plaque formation, leading to neuron cell death^6,7^. LPS-induced neuroinflammation increases β-secretase and γ-secretase expression in mice^43^. Moreover, the inhibition of β-secretase and γ-secretase expression and induction of BDNF expression by *Lactobacillus plantarum* C29 treatment alleviates memory impairment in 5XFAD-Tg mice^19^. These results suggest that NK46 can improve cognitive function in 5XFAD-Tg and aged mice by increasing BDNF expression and suppressing NF-κB activation and β-secretase and γ-secretase expression, which could be both dependently and independently induced by the Aβ formation. NK46 significantly suppressed LPS-induced NF-κB activation in *in vitro* study. Furthermore, NK46 suppressed gut microbiota LPS-induced NF-κB activation in the colon and hippocampus. These results suggest that NK46 and its byproducts such as lipoteichoic acids and short chain fatty acids may inhibit gut dysbiosis and bacterial LPS production, resulting in the attenuation of the gut inflammation. Overall, NK46 alleviated gut dysbiosis, colitis, and cognitive decline in 5XFAD-Tg mice more potently in aged mice. These results may be due to short-term (4 weeks) treatment with NK46 in aged mice compared to its treatment for 8 weeks in 5XFAD mice.

Conclusively, our finding support the suggestion that gut dysbiosis and their excessive endotoxin production may cause endotoxemia and systemic inflammation, resulting in neuropsychiatric disorders: neuropsychiatric disorder-inducible stressors can alter gut dysbiosis through the regulation of HPA axis and gut dysbiosis can cause psychiatric disorders through the regulation of MGB axis^44–46^. Moreover, *Bifidobacteirum longum* can modify gut microbiota, particularly the Proteobacteria population, and their LPS production in 5XFAD-Tg and aged mice and suppress the progression of GI inflammation and endotoxemia, resulting in the attenuation of cognitive decline in 5XFAD-Tg and aged mice with the regulation of neuroinflammation via MGB axis.

## Materials and Methods

### Materials

Antibodies for p65, p-p65, BDNF, claudin-5, occludin, caludin-1, iNOS, COX-2, and β-actin were purchased from Cell Signaling Technology (Beverly, MA). Enzyme-linked immunosorbent assay (ELISA) kits for cytokines were purchased from Ebioscience (San Diego, CA). 4′,6-Diamidino-2-phenylindole dilactate (DAPI) was purchased from Sigma (St. Louis, MO). QIAamp Fast DNA stool mini kit was purchased from Qiagen (Hilden, Germany). Limulus amoebocyte lysate (LAL) assays was purchased from Cape Cod Inc. (East Falmouth, MA). General anaerobic medium (GAM), BL, and DHL media were from Nissui Pharmaceutical Co. (Tokyo, Japan). MRS medium was purchased from BD (Radnor, PA).

### Isolation of fecal microbiota LPS production-inhibitory gut bacteria

Mouse fresh stools were diluted with GAM broth and centrifuged (500 g, 5 min). The supernatant (1 × 10^5^ colony forming units [CFU]) was anaerobically cultured at 37 °C for 24 h with Lactobacilli or Bifidobacteria (1 × 10^5^ CFU or 1 × 10^6^ CFU) in GAM broth, sonicated for 1 h on ice, centrifuged (400 g, 10 min), filtered through a 0.45-μm filter, and refiltrated through a 0.22-μm filter^19^. Supernatant LPS levels were measured using an LAL Assay Kit.

### Culture of *Bifidobacterium longum*

*Bifidobacterium longum* (NK46) was selected from the collection of human fecal Bifidobaceria and Lactobacilli strains as the LPS production bacterium. NK46 was cultured in in MRS broth (2L), centrifuged (5,000 *g*, 20 min), and washed with saline. Cells (1 × 10^9^ CFU/0.1 mL) were suspended in phosphate-buffered saline (for *in vitro* experiment) or 1% glucose (for *in vivo* experiment).

To decide the dose of NK46 in mouse experiments, NK46 (1 × 10^8^ and 1 × 10^9^ CFU/mouse/day) were orally treated for 5 days in immobilization stress-treated mice and its anti-colitis effects such as colon length and macroscopic score were measured according to the method of Lee *et al*.^19^. Treatment with NK46 at a dose of 1 × 10^9^ CFU/mouse/day alleviated more potently than at a dose of 1 × 10^8^ CFU/mouse/day. Therefore, we orally gavaged NK46 at a dose of 1 × 10^9^ CFU/mouse/day for the further *in vivo* study.

### Culture of BV-2 cells

BV-2 cells were cultured at 37 °C in a 5% CO_2_–95% air in DMEM containing 1% antibiotic-antimycotic and 5% fetal bovine serum according to the method of Lee *et al*.^47^. BV-2 cells (1 × 10^6^ cells/mL) were stimulated with LPS (100 ng/mL) in the presence or absence of NK46 (1 × 10^5^ CFU/mL) for 90 min. NF-κB activation (p-p65/p65) was measured by immunoblotting.

### Animals

All animal experiments were approved by the Committee for the Care and Use of Laboratory Animals in the Kyung Hee University (KHUASP(SE) 17–029 and 17–128) and were performed according to the NIH and Kyung Hee University Guidelines for Laboratory Animals Care and Use. Mice were fed a standard laboratory diet, allowed to take water ad libitum, and maintained in a ventilated room (temperature, 22 °C ± 1 °C; humidity, 50% ± 10% humidity); and a 12-h diurnal light cycle, 07:00–19:00) for 2 months before the animal experiment. They were housed in wire cages (3 mice/cage).

Male B6SJL-Tg (APPSwFlLon, Psen1*M146L*L286V) 6799Vas/J transgenic (5XFAD) mice (4-months-old) were supplied from Jackson Laboratories (Bar Harbor, ME). Male C57BL/6 mice (4-months- and 16-months-old) were supplied from Raonbio Inc. (Yongin, Gyunggi-do, Korea).

5XFAD-Tg mice (6 months-old) were separated into two groups, which were treated with vehicle or NK46 (1 × 10^9^ CFU/day/mouse) six times per week for 8 weeks, as previously reported^47^. Control mice (6 months-old) were treated with vehicle. Each group consisted of six mice.

Aged mice (18 months-old) were also separated into two groups, which were treated with vehicle or NK46 (1 × 10^9^ CFU/day/mouse) six times per week for 4 weeks. Control mice (6 months-old) were treated with vehicle for 4 weeks. Each group consisted of six mice.

For the assays of biochemical parameters, mice were then anesthetized 2 h after performing the final task. Blood, brain and colon were removed. Plasma was prepared by centrifuging blood. Colons were opened longitudinally and gently cleared of stool using phosphate-buffered saline (PBS). These tissues were used for immunostaining, immunoblotting, and ELISA.

### Memory behavioral tasks

Y-maze task was performed according to the method of Jang *et al*.^48^. Passive avoidance task was performed according to the method of Jung *et al*.^26^. Novel object recognition task was performed in the apparatus consisted of a dark-open field box (45 × 45 × 50 cm) according to the method of Lee *et al*.^19^. Morris water maze task was performed according to the method of Jung *et al*.^26^.

### Immunofluorescence assay

Immunostaining analysis of brain slices was performed according to the method of Duncan and Miller^49^ and Jang *et al*.^22^. Microglial cells were visualized by staining with anti-Iba1 antibody (1:200, Santa Cruz). Apoptotic neuron cells were stained with anti-caspase-3 and anti-NeuN antibodies (1:500, Millipore). LPS were stained with ant-LPS antibody (1:200, Abcam). Briefly, the brains were cryoprotected in 30% sucrose-PBS and then frozen with optimal cutting temperature compound and stored at −80 °C until processed. Brain tissue blocks were cryosectioned at a thickness of 30 μm, stored at 4 °C in the storing solution (30% ethylene glycol in PBS), permeabilized in 0.5% Triton X-100 for 5 min, blocked in 10% bovine serum with tween 20-contained PBS for 30 min, and incubated for 16 h at 4 °C with antibodies. Secondary antibodies conjugated with Alexa Fluor 488 (1:1,000, Invitrogen) or Alexa Fluor 594 (1:500, Abcam) were then treated to visualize. Nuclei were stained with DAPI. Immunostained samples were scanned with a confocal laser microscope.

### Immunoblotting

Brain and colon tissues and cultured cells were homogenized with RIPA lysis buffer containing 1% protease inhibitor cocktail and a phosphatase inhibitor cocktail on the ice and centrifuged (13,200 *g*, 10 min, 4 °C)^19,48^. Proteins of supernatants were electrophoresed on sodium dodecyl sulfate-polyacrylamide gel, transferred to nitrocellulose membrane, blocked with non-fat dried-milk proteins, probed with antibodies for BDNF, CREB, p-CREB, p65, p-p65, COX-2, p16, caspase-3, Aβ, and β-actin, washed with PBS containing tween 20, and treated with secondary antibodies conjugated with horseradish peroxidase. Protein bands were visualized with an enhanced chemiluminescence detection kit.

### Myeloperoxidase activity assay

Colon tissues were homogenized in 10 mM potassium phosphate buffer (pH 7.0) containing 0.5% hexadecyl trimethyl ammonium bromide, and centrifuged (13,200 *g*, 10 min, 4 °C). The resulting supernatants (50 μL) were added to the reaction mixture containing 0.1 mM H_2_O_2_ and 1.6 mM tetramethyl benzidine preincubated at 37 °C for 2 min, and sequentially monitored the absorbance (650 nm) at 37 °C for 5 min^48^. Myeloperoxidase activity was calculated as the quantity of enzyme degrading 1 μmol/mL of peroxide, and expressed in unit/mg protein.

### Determination of LPS

Blood and fecal LPS levels were measured according to the methods of Kim *et al*.^50^. For the assay of blood LPS contents, bloods collected by retroorbital bleeding into ethylenediaminetetraacetic acid-coated BD Microtainer^®^ tubes (Becton Dickinson, Franklin Lakes, NJ, USA) were centrifuged (13,200 *g*, 15 min). The supernatant (5 μL) was diluted 1:10 in pyrogen-free water and inactivated for 10 min at 70 °C. For the assay of fecal LPS contents, mouse feces were placed in 50 mL of PBS in a pyrogen-free tube, sonicated for 1 h on ice, and centrifuged (400 *g*, 10 min). The supernatant was collected, filtrated through a 0.45 μm Millipore filter, re-filtrated through a 0.22 μm filter, and inactivated at 70 °C for 10 min. Each filtrate or supernatant (50 μL) was incubated with LAL solution at 37 °C for 30 min, added additional reagents to formation of a magenta derivative, and measured the absorbance at 545 nm.

### Culture of fecal bacteria

Fecal Enterobacteriaceae and Lactobacilli/Bifidobacteria populations were counted using the selective media, DHL and BL agar plates, according to the method of Kim *et al*.^50^.

### Pyrosequencing

DNA was extracted from the fresh stools of mice (excluded mice trans-cardiacally perfused for brain tissue fixation) using a commercial DNA isolation kit (QIAamp DNA stool mini kit), as previously reported^20^. Genomic DNA was amplified using barcoded primers, which targeted the V3 to V4 region of the bacterial 16S rRNA gene. Pyrosequencing was carried out using a 454 GS FLX Titanium Sequencing System (Roche, Branford, CT) according to the method of Lee *et al*.^19^. Sequence reads were identified using the EzTaxon-e database (http://eztaxon-e.ezbiocloud.net/) on the basis of bacterial 16S rRNA sequence data. The number of sequences analyzed, observed diversity richness (operational taxonomic units, OTUs), estimated OTU richness (ACE and Chao1), and coverage were calculated using the Mothur program and defined considering a cut-off value of 97% similarity with the bacterial 16S rRNA gene sequences. Pyrosquencing reads have been deposited in the NCBI’s short read archive under accession number SRX3921564~3921588.

### Statistical analysis

Experimental data are indicated as means ± standard deviation, and were statistically analyzed using one-way ANOVA followed by Duncan’s multiple range test (*P* < 0.05).
